# Neuromorphic engineering in wetware: the state of the art and its perspectives

**DOI:** 10.3389/fnins.2024.1443121

**Published:** 2024-09-10

**Authors:** Pier Luigi Gentili, Maria Pia Zurlo, Pasquale Stano

**Affiliations:** ^1^Department of Chemistry, Biology, and Biotechnology, Università degli Studi di Perugia, Perugia, Italy; ^2^Department of Biological and Environmental Sciences and Technologies (DiSTeBA), University of Salento, Lecce, Italy

**Keywords:** chemical artificial intelligence, chemical reaction networks, emergence, oscillatory chemical reactions, synthetic biology, DNA, proteins, fluidic memristors

## 1 Introduction

**Figure 1:**
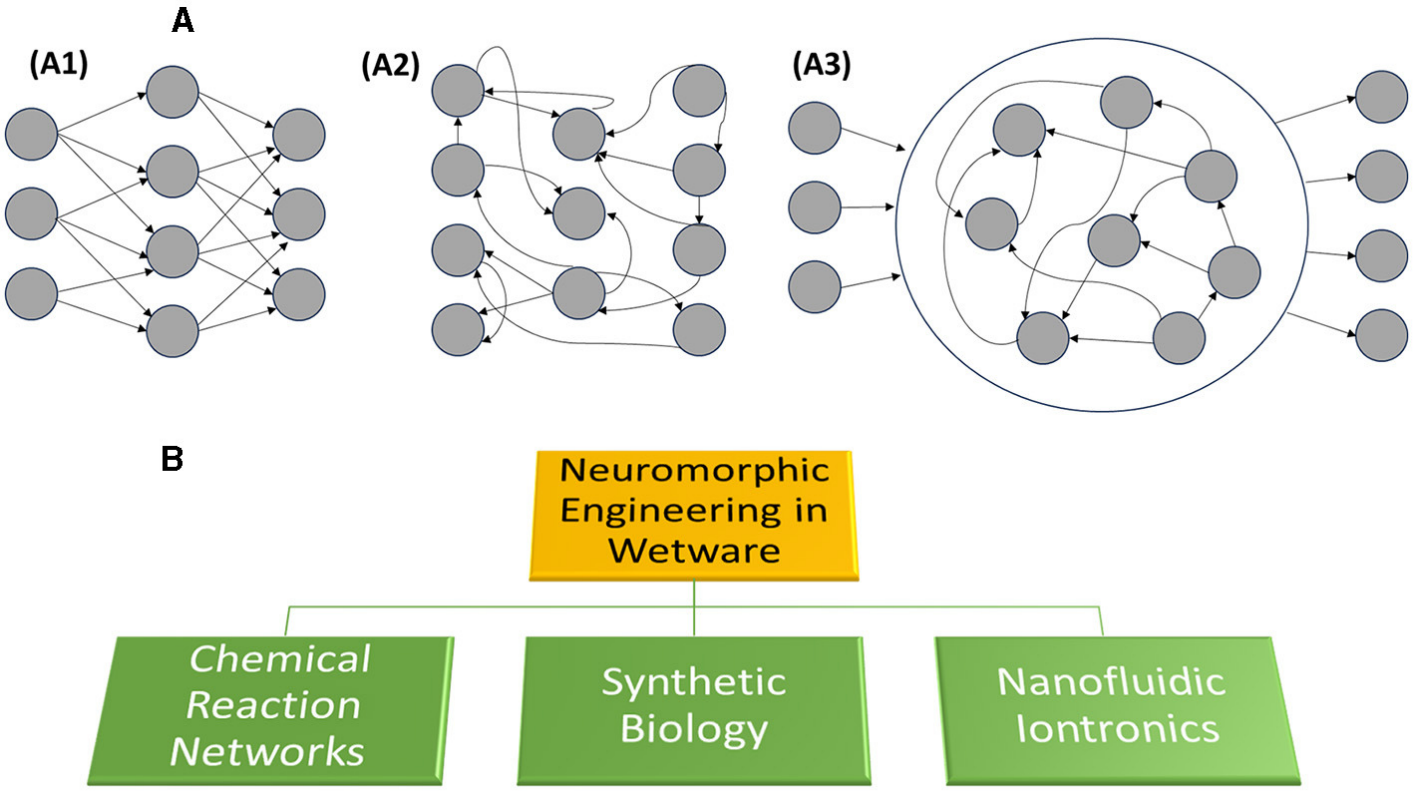
**(A)** Shows the three principal architectures of artificial neural networks: they are (A1) feedforward, (A2) recurrent, and (A3) reservoir networks. **(B)** Showcases the three principal methodologies for developing neuromorphic engineering in wetware.

The UN General Assembly (2015) has compiled an Agenda, containing 17 goals to be pursued worldwide to promote a sustainable future by 2030. Accomplishing these goals requires designing and implementing more effective strategies to manage Complex Systems, including human beings and their societies, the world economy, urban areas, natural ecosystems, and the climate (Gentili, 2021a). A promising strategy, which is literally blooming, relies on the development of Artificial Intelligence (AI) and Robotics. AI helps humans collect, store, and process the Big Data required to monitor the constant evolution of Complex Systems (Corea, 2019). AI also assists us in making up our minds for controlling the behavior of Complex Systems. Hard and soft robotics allow humans to access environments otherwise precluded. For instance, they help us (1) investigate the geochemical characteristics of other planets and examine the abysses of our oceans to discover new mines of precious materials and energy resources, (2) access the interior organs of our bodies for less invasive surgery, (3) and work in dirty or dangerous places. Two are the principal and traditional approaches exploited to develop AI (Lehman et al., 2014; Mitchell, 2019). The first approach entails writing “intelligent” software that runs on electronic computers based on von Neumann's architecture, whose principal drawback is having processing and memory units physically separated. Some software mimics rigorous logical thinking, while others imitate the structural and functional features of neural networks to learn how to perform tasks from data. The second approach for developing AI entails implementing artificial neural networks in hardware for neuro-prosthesis or designing brain-like computing machines, with processors and memory confined in the same space (the so-called mem-computing; Sebastian et al., 2020). Artificial neural networks are rigid if they are made of silicon-based circuits or inorganic memristors; they are flexible if based on organic semiconductor films (Christensen et al., 2022; Lee and Lee, 2019; Wang et al., 2020; Zhu et al., 2020). They can be designed with three distinct architectures: (A1) feedforward (having trainable unidirectional connections), (A2) recurrent (with trainable feedback actions), or (A3) reservoir (consisting of an untrained non-linear dynamic system coupled to trainable input and output layers) network (Nakajima, 2020; Tanaka et al., 2019; Cucchi et al., 2022; see Figure 1A).

In the last decade or so, a novel promising strategy to develop AI has been put forward: it consists of mimicking human intelligence and the forms of intelligence exhibited by all the other living beings through molecular, supramolecular, and systems chemistry in wetware, i.e., liquid solutions (Gentili and Stano, 2023a,b; Kuzuya et al., 2023; Murata et al., 2022), which is the peculiar phase supporting life. As we believe that this still not-well-explored field represents a huge opportunity to understand and exploit computation in the molecular realm—thus closely mimicking the natural (biological) cognitive abilities—here we would like to highlight the current methodologies. In particular, we focus on artificial neural networks in wetware and, hence, on the strategies to develop neuromorphic engineering in the fluid phase. The selection of topics presented in this short article is not meant to represent the whole diversity of this research area—it rather mirrors our specific interests. The variegate methodologies proposed so far can be grouped into three distinct approaches (see Figure 1B) presented succinctly in the next three paragraphs. Some future perspectives are shortly presented in the last paragraph.

## 2 Chemical reaction networks

Any liquid solution containing two or more reactive solutes may display some of the brain's dynamic features, especially if considered as a useful model or even a simplified version of it. Although any brain is a complex three-dimensional cellular architecture, chemical reaction networks can share some aspects of their organization. Indeed, it is still possible to draw direct analogies between the chemical compound and chemical reaction space to bio-inspired brain-type architectures with the reactive molecules of solutes representing the neurons and their mutual impacts being the synapses (Csizi and Lörtscher, 2024). The molecules of solvent, which do not react but assist the chemical transformations of solutes, are like the brain's glial cells. Some solute molecules' collisions trigger chemical reactions, whereas others are chemically ineffective. Specific steric and energetic conditions must be verified to render a molecular impact reactive. The Arrhenius law defines the transformation rate constant (*k*_*r*_) of the reagents into the products and it formally corresponds to the activation function of the molecular nodes:


(1) $$\begin{array}{l} k_{r}=Ae^{\frac{-E_{act}}{RT}} \end{array}$$

In Equation 1, the pre-exponential factor *A* is related to the steric requirements, whereas *E*_*act*_ is the minimum energy needed to make an impact reactive. Usually, it is the thermal energy, *RT*, available to all the molecules, which is exploited to overcome the barrier *E*_*act*_, unless other energetic inputs are unleashed from outside. The kinetic constant *k*_*r*_, defined in Equation 1, is related to the computational rate for the chemical reaction network: it increases by heating. If the concentration of *i*-th solute is *C*_0, *i*_ (expressed in moles per volume of solution, i.e., in molarity *M*), the total number of molecular neurons (*N*) per unit of volume (expressed in liters) will be given by the Avogadro's number times the sum of the solutes' analytical concentrations:


(2) $$\begin{array}{l} N=\left(6.022\times 10^{23}\right)\underset{i}{\sum }C_{0,i} \end{array}$$

Molecular networks compute in a highly parallel manner, and their computational rate (*C*_*R*_) might be remarkable: For a bimolecular reaction of the type $A+B\overset{k_{r}}{\to }P$, it will be:


(3) $$\begin{array}{l} C_{R}=\left(k_{r}C_{0,A}C_{0,B}\right)\left(6.022\times 10^{23}\right) \end{array}$$

When the rate-determining step is the encounter of the reactants (A and B) by diffusion, the apparent reactive constant ${\left(k_{r}\right)}_{app}\approx 10^{9}M^{-1}s^{-1}$, and if $C_{0,A}C_{0,B}\approx 10^{-9}M^{2}$, then the computational rate is hundreds of zettaFLOPS (i.e., ≈10^23^) per unit of volume, i.e., five orders of magnitude faster than the best supercomputer in the world, according to the TOP500 project (https://www.top500.org/). Of course, in a chemical reaction, even if carried out by billions and billions of molecules, it is generally impossible to address individual reaction events in order to distinguish them because they occur randomly distributed in space and time. The situation could be improved through micro-compartmentalization, but it remains far from the performances of the two-dimensional architecture of the processors inside an electronic computer and even further from the remarkable computational performances of the three-dimensional architecture of a biological brain.

In any fluid solution, the network's architecture is not fixed, but fluid, subjected to the constant movement of the molecular neurons, promoted by diffusion, stirring (if present), and advection (if induced). It is a reservoir network (Figure 1A3), whose overall shape and size are fixed by the solid device containing the solution (Adamatzky, 2019) and whose computational rate is directly proportional to the concentrations of the solutes. If the molecules constituting the network are prepared and maintained in a coherent quantum state, they can be employed to perform quantum neuromorphic computing (Ghosh et al., 2021). When molecular Brownian motion destroys the coherent quantum states, the chemical reservoir can be exploited to implement classical logic. If the input-output relationships are steep sigmoid functions, they are appropriate for implementing binary logic gates (De Silva, 2013). The molecular logic gates have been demonstrated to be reconfigurable because the input-output relationship can change depending on the technique used to monitor the read-out layer of Figure 1A3. When the input-output function is not sigmoid but hyperbolic or linear, the molecular network is appropriate for processing infinite-valued logic, like fuzzy logic (Gentili, 2018). Fuzzy logic is a model of human capability to make decisions using natural language. The words are fuzzy sets. It has been demonstrated that fuzzy sets can be chemically implemented through the context-dependent conformational distributions of compounds (Gentili, 2021b; Gentili and Perez-Mercader, 2022). The major challenges for neuromorphic engineering through chemical reaction networks are to connect (1) different chemical logic gates for the implementation of extended circuits analogous to those in electronics and (2) distinct chemical words to build molecular languages. One way is through optical signals (Andréasson and Pischel, 2015) and another through microfluidic platforms that allow controlling the encounter of molecular reagents (Kou et al., 2008).

Some chemical reactive systems produce intermediates that establish mutual strong non-linear relationships, typical of a recurrent network, and give rise to bottom-up emergent properties, such as spontaneous temporal and spatial self-organization phenomena (Epstein and Pojman, 1998; Ashkenasy et al., 2017). These chemical systems, whose iconic instance is the Belousov-Zhabotinsky reaction, have been proposed as dynamic surrogates of real neurons because they can reproduce their oscillatory, chaotic, and excitable regimes (Okamoto et al., 1995; Izhikevich, 2007; Gentili and Micheau, 2020). They can communicate through chemical, electrical, and optical signals, giving rise to spatio-temporal synchronization phenomena, analogous to those shown by real neural networks. The single neural surrogates can be confined to either macro- or micro-reactors. They have been arranged in all three archetypes of neural networks shown in Figure 1A: feed-forward, recurrent, and reservoir networks (Gentili et al., 2017; Litschel et al., 2018; Vanag, 2019; Gentili, 2022; Tomassoli et al., 2024).

When the molecules participating in the chemical reaction networks are biopolymers, such as DNA, RNA, and proteins, we enter the realm of synthetic biology, which constitutes the second strategy for developing neuromorphic engineering in wetware (Vasle and Moškon, 2024).

## 3 Synthetic biology

The non-linear reactivity of biopolymers, i.e., DNA, RNA, and proteins engaged in fundamental processes for cell life, is ideal for implementing reservoir and recurrent networks (Cameron et al., 2014; Tang et al., 2021) *in vivo* and *in vitro*. Since each biopolymer exists as a collection of conformers, whose features are context-dependent, the bio-chemical reaction networks are intrinsically fuzzy (Gentili, 2024). Fuzzy neural networks guarantee adaptability and the capability to make decisions in environments dominated by uncertainty and vagueness (Zadeh, 1997; Gentili and Stano, 2022). Within a cell, biopolymers participate in chemical reactions that occur in overcrowded micro- and nano-compartments (i.e., the organules), often at their interface, and involving tethered reactive species, limiting their random Brownian motions. This well-orchestrated and complex bio-chemical reaction network gives rise to an autonomous cellular computing system. A cell is capable of (1) collecting data about the external environment and its internal state through transmembrane sensory proteins; (2) processing the sensory data and making decisions, which (3) trigger the genetic module or (4) modify cellular metabolism (Roederer, 2005; Gentili and Stano, 2024). Living cells are too complex to be reproduced synthetically, from scratch, through a bottom-up approach. The synthetic cells (SCs) implemented so far are more similar to wetware machines that are programmed to compute and accomplish specific tasks, such as assaying chemical information and therapeutics (Chang, 1987; Guindani et al., 2022). However, attention has been recently paid to how to make them more organism-like, i.e., “minimally cognitive” (Damiano and Stano, 2018; Stano, 2023). For example, an explicitly declared goal is to implant a sort of minimal brain made of chemical reaction networks inside SCs (Braccini et al., 2023), aiming at a simple form of autonomy. A step further will be reachable when an SC could become a neural network node made of other SCs (with or without involving natural cells) to imitate the organizational and functional features of biological tissues. In these cellular networks, two- or three-dimensional cultures of human brain cells (the so-called brain organoids) will be employed, facilitating the reconstruction of the histoarchitecture and functionality of real neural networks (Smirnova, 2024).

## 4 Nanofluidic iontronics

Bioinspired nanofluidic iontronics represents the most recent approach for developing neuromorphic engineering in wetware (Hou et al., 2023). It consists of hybrid circuits made of solid nanochannels and electrically conductive ionic solutions to imitate real neurons that use ionic currents as information carriers. The solid nanochannels are not simple containers: their shape and size affect the electrical properties of the devices. There are two groups of nanofluidic devices: (1) nanofluidic transistors that mimic structures and functionalities of biological ion channels, and (2) nanofluidic memristors that mimic synapses (Xiong et al., 2023a). Under nano-confinements, both water molecules and ions exhibit anomalous transport behaviors, such as ultrahigh ion/proton transport speed and selectivity (Robin et al., 2023). These nanofluidic devices not only reproduce brain-like neural electrical signals but also realize the logic operation or memory functionalities. The way to endow bioinspired nanofluidics with smart responsiveness is to modify the inner surface of the channels with various responsive molecules, such as aptamers and antibodies (Xiong et al., 2023b). A wide range of chemical species could coexist and move freely in electrolyte solutions contributing to abundant chemical information compared with solid memristors. The biological compatibility of fluidic memristors is convenient for the communication between real neurons and devices.

## 5 Discussion

Despite the recent impressive advancements in conventional (hardware/software) AI and Robotics, we expect a profound revolution in the sciences of the artificial (Cordeschi, 2002) will definitely come from exploring fluid chemical systems and their computational capabilities. The development of neuromorphic engineering in wetware requires an interdisciplinary effort, involving chemists, physicists, biologists, engineers, computer scientists, and neuroscientists. Differently from general-purpose electronic computers, neuromorphic devices in wetware will be specific-purpose. In computing, they will be particularly alluring for recognizing variable patterns, solving NP-hard problems, and processing vague information (Adleman, 1994; Adamatzky et al., 2005; Evans et al., 2024; Csaba and Porod, 2020; Gentili and Stano, 2024) because chemical reaction networks perform massive-parallel computations. Furthermore, neuromorphic devices in wetware will guarantee a seamless interface with living beings because they can interplay with living cells even at the molecular level. They will reciprocally communicate through both chemical and physical signals. Chemical communication can be carried out not only through diffusion, but also advection, chemical waves and motor proteins. Neuromorphic devices in wetware will monitor, and heal if required, biological functions through the implementation of multi-scale artificial and biological communication networks, called Internet of Nano/Bio-things (IoBNTs; Akyildiz et al., 2015; Stano et al., 2023). We think it is reasonable to expect that such IoBNTs will approach the power of biological intelligence to process information based on uncertain and context-dependent data without an excessive expenditure of energy.
